# Modifications of the mechanical properties of *in vivo* tissue-engineered vascular grafts by chemical treatments for a short duration

**DOI:** 10.1371/journal.pone.0248346

**Published:** 2021-03-12

**Authors:** Tomoya Inoue, Keiichi Kanda, Masashi Yamanami, Daisuke Kami, Satoshi Gojo, Hitoshi Yaku

**Affiliations:** 1 Department of Cardiovascular Surgery, Graduate School of Medical Science, Kyoto Prefectural University of Medicine, Kyoto, Japan; 2 Department of Regenerative Medicine, Graduate School of Medical Science, Kyoto Prefectural University of Medicine, Kyoto, Japan; Politecnico di Milano, ITALY

## Abstract

*In vivo* tissue-engineered vascular grafts constructed in the subcutaneous spaces of graft recipients have functioned well clinically. Because the formation of vascular graft tissues depends on several recipient conditions, chemical pretreatments, such as dehydration by ethanol (ET) or crosslinking by glutaraldehyde (GA), have been attempted to improve the initial mechanical durability of the tissues. Here, we compared the effects of short-duration (10 min) chemical treatments on the mechanical properties of tissues. Tubular tissues (internal diameter, 5 mm) constructed in the subcutaneous tissues of beagle dogs (4 weeks, n = 3), were classified into three groups: raw tissue without any treatment (RAW), tissue dehydrated with 70% ET (ET), and tissue crosslinked with 0.6% GA (GA). Five mechanical parameters were measured: burst pressure, suture retention strength, ultimate tensile strength (UTS), ultimate strain (%), and Young’s modulus. The tissues were also autologously re-embedded into the subcutaneous spaces of the same dogs for 4 weeks (n = 2) for the evaluation of histological responses. The burst pressure of the RAW group (1275.9 ± 254.0 mm Hg) was significantly lower than those of ET (2115.1 ± 262.2 mm Hg, p = 0.0298) and GA (2570.5 ± 282.6 mm Hg, p = 0.0017) groups. Suture retention strength, UTS or the ultimate strain did not differ significantly among the groups. Young’s modulus of the ET group was the highest (RAW: 5.41 ± 1.16 MPa, ET: 12.28 ± 2.55 MPa, GA: 7.65 ± 1.18 MPa, p = 0.0185). No significant inflammatory tissue response or evidence of residual chemical toxicity was observed in samples implanted subcutaneously for four weeks. Therefore, short-duration ET and GA treatment might improve surgical handling and the mechanical properties of *in vivo* tissue-engineered vascular tissues to produce ideal grafts in terms of mechanical properties without interfering with histological responses.

## Introduction

With population aging and an increase in the number of patients undergoing long-term dialysis, the frequency of re-operative surgeries, such as repetitive coronary bypass grafting [1] and distal bypass operation of the lower limb below the knee [2] are on the rise. Various vascular prostheses have been developed; however, substitute blood vessels that can be used for these kinds of bypass surgeries are currently only autologous arteries and veins. Because autologous vessels might have already been used for the other kinds of bypass grafts or dialysis shunts, autologous vascular grafts could be insufficient during repetitive surgeries. Therefore, the development of a substitute blood vessel for a small caliber arterial bypass or dialysis shunt is an urgent need. In artificial material development and artificial surface processing technology, it is difficult not only to impart long-term antithrombotic property but also to prevent anastomotic site intimal thickening or pannus formation resulting in anastomotic stenosis causing late-stage obstruction. Rapid and efficient organization by autologous vascular wall cells is the only way to avoid these shortcomings [3, 4].

It has long been known that when a foreign object is implanted within the body, it is covered with a capsule-like tissue, thereby forming a biological defense mechanism [5]. This phenomenon is controlled by the latest material engineering technology to develop small caliber substitute blood vessels called "Biotubes," consisting of only autologous tubular connective tissues constructed by *in vivo* tissue engineering [6]. These are inexpensive and easily manufactured without the need for complicated procedures or specialized clean manufacturing facilities. Additionally, Biotubes function as superior autologous small diameter vascular grafts to the synthetic prostheses, such as rapid endothelialization and hierarchical tissue regeneration mimicking native arterial wall structures in animal models [7, 8]. Moreover, Biotubes are successfully applied clinically during pulmonary arterial patch plasty in pediatric patients [9].

However, because individual differences in tissue formation exist, it is sometimes difficult to reliably produce tissues that can withstand mechanical stress. Particularly during clinical applications, overcoming individual variations is a major issue as recipients are a diverse population, such as young children, the elderly, diabetic patients, and dialysis patients with extremely poor tissue healing ability. Therefore, several kinds of chemical pretreatments such as dehydration by ethanol (ET) or crosslinking by glutaraldehyde (GA) have been attempted to modify the mechanical properties of engineered tissues for producing ideal grafts from mechanical aspects. In this study, we compared the effects of such treatments on the mechanical properties of *in vivo* tissue-engineered vascular grafts.

## Materials and methods

### Preparation of tubular connective tissues

The experimental procedures and the study protocol (no. M29-26) were approved by the Animal Experiment Ethics Committee of the Kyoto Prefectural University of Medicine (Kyoto, Japan). The experiment was performed in accordance with the US Animal Welfare Act.

**Fig 1:**
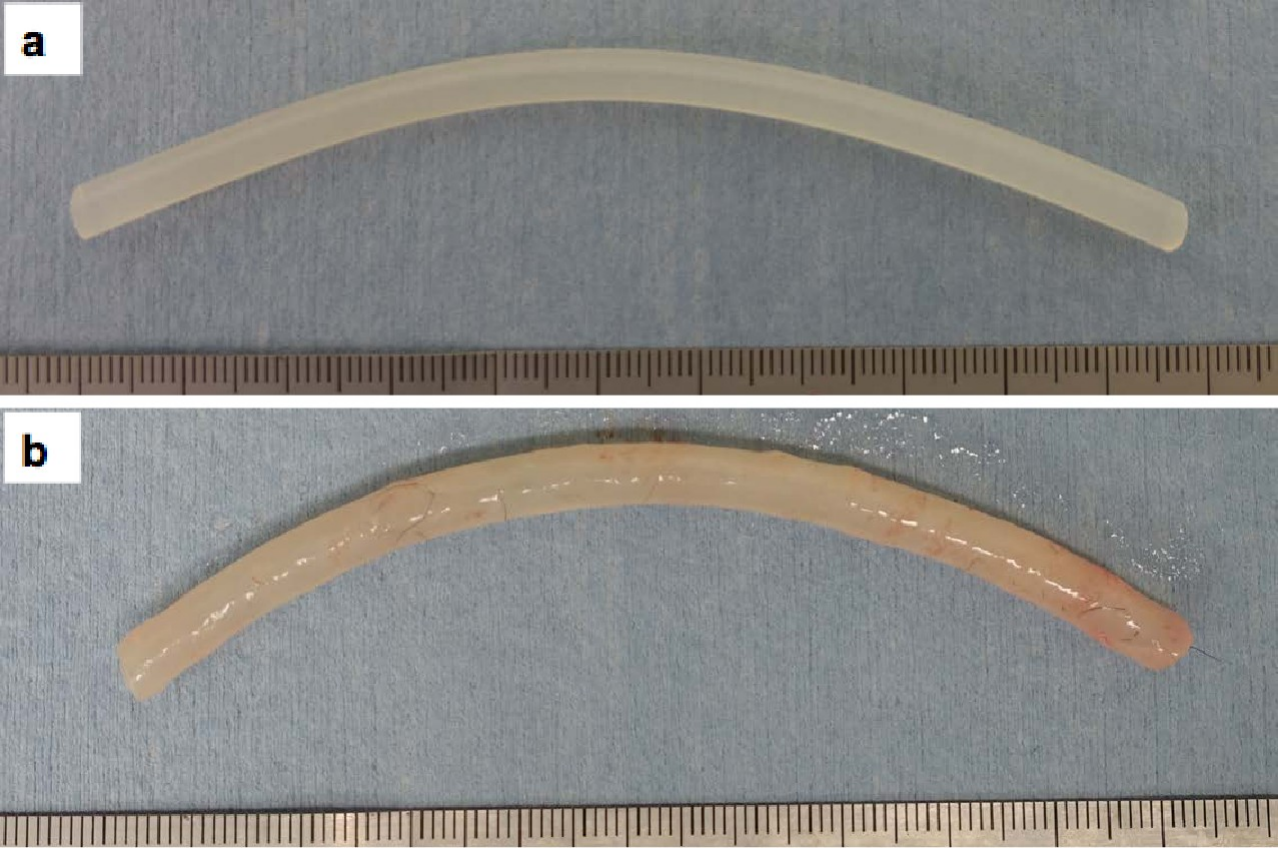
Silicone rod mold (a) and constructed *in vivo* tissue-engineered vascular graft (b).

Tubular connective tissues were constructed according to a previous study [7]. Briefly, silicone rods of 5 mm in outer diameter (the outer diameter of the silicone rod equals the inner diameter of the tissue because the tissue was constructed around the silicone rod) and 10 cm in length (3-2316-01, AS ONE, Osaka, Japan) were used as molds (Fig 1A). One-year-old female beagle dogs (n = 3) purchased from OrientalBioService (Kyoto, Japan) were anesthetized with intramuscular ketamine (20 mg/kg) and xylazine (0.1 mg/kg). Small incisions were made in the shaved dorsal skin, and nine molds were placed within dorsal subcutaneous pouches of each dog. After four weeks, the dogs were anesthetized, and the implants were harvested. After removing the inner silicone rod molds 5 mm in outer diameter, the connective tissue tube grafts 5 mm in inner diameter and 10 cm in length were acquired (Fig 1B). The dogs were euthanized by high-dose pentobarbital after the experiment.

### Chemical modifications of the tubular tissues

Both ends of the connective tissue tubes were trimmed 5 mm in length, and the remaining tubular tissues of 9 cm length were cut into three pieces (3 cm in length), which were randomly divided into three groups: an untreated group kept in saline (RAW group), a treated group immersed with 70% ET for 10 min (ET group), and a treated group immersed with 0.6% GA for 10 min (GA group) at room temperature (25°C). After each treatment, the tissues were rinsed with saline thrice for 3 min to wash out the remnant chemicals. To prevent drying out, all samples were stored in saline before assessment.

### Vessel ringlet pull test

**Fig 2:**
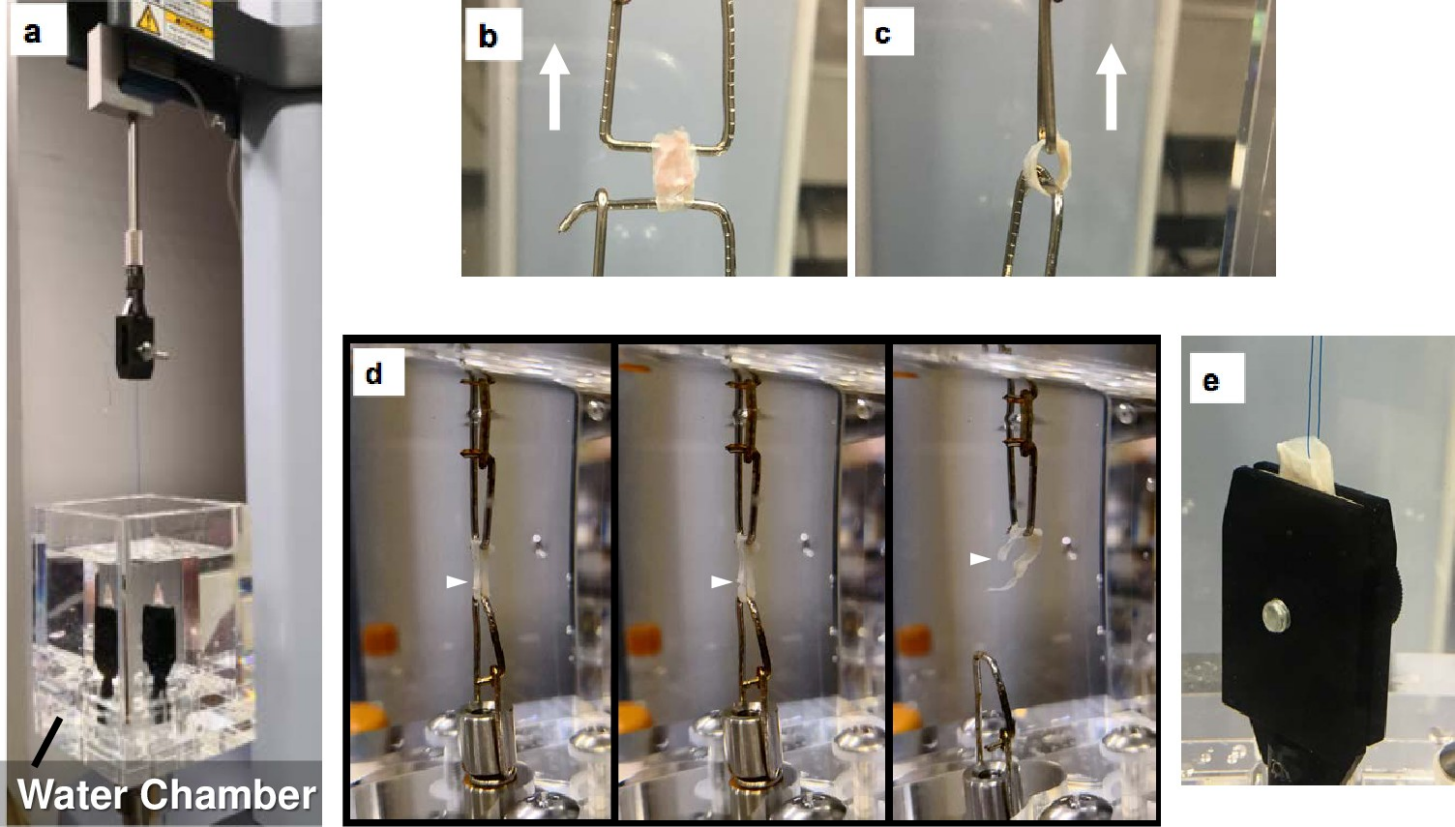
The entire appearance of the system including the specially designed water chamber for vessel ringlet pull test and suture retention stress test (a). A vessel ringlet sample was held by two parallel hooks (b: front view, c: side view). A broken sample by vessel ringlet pull test (d). White triangles indicate failure points. A sample for suture retention testing held in saline by the test piece grasping device. A 5–0 prolene suture was placed at the top end of the tissue tube (e).

Vessel ringlet pull test was performed as specified in ISO 7198 [10] in a specially designed water chamber (Fig 2A) filled with saline to maintain the wet conditions. Tissue tube segments of 5 mm in length (n = 18 for each treatment) were placed around two parallel hooks made of stainless steel wire of 1 mm in diameter (Fig 2B and 2C). The actual lengths of the tissue tubes were measured by a digital vernier caliper to convert the value to 5 mm equivalence. The hooks were then pulled apart at a rate of 100 mm/min and the force curve was recorded by a uniaxial tensile tester (Shimadzu EZ-SX 100N, Kyoto, Japan) until failure. The experiments were photographed to verify that failure occurred in the middle of the tissue away from the hooks (Fig 2D). From the recorded force curve, three kinds of mechanical parameters were determined: ultimate tensile strain (UTS) [maximal tension divided by a cross-sectional area of tissue during the recording (MPa = N/mm^2^)], ultimate strain [elongated length at the maximal tension divided by the initial length (%)], and Young’s modulus [maximal slope from the stress-strain curve (MPa)].

### Suture retention testing

Suture retention tests were also conducted as specified in ISO 7198. The bottom end of the tissue tube segment (10 mm long) (n = 6, for each treatment) was clamped by a test piece grasping device. A single 2 mm bite of 5–0 prolene suture with BB-1 needle (Ethicon Inc. Ohio, USA) was placed at the top end of the tissue tube (Fig 2E) and pulled up at a constant speed of 100 mm/min. The force curve was recorded until the tissue failed in the wet conditions using the same apparatus used for vessel ringlet pull test as mentioned above. The test was repeated twice on the same sample at locations 120° apart to obtain three readings for each vessel test segment (n = 18 for each treatment).

### Burst pressure measurements

**Fig 3:**
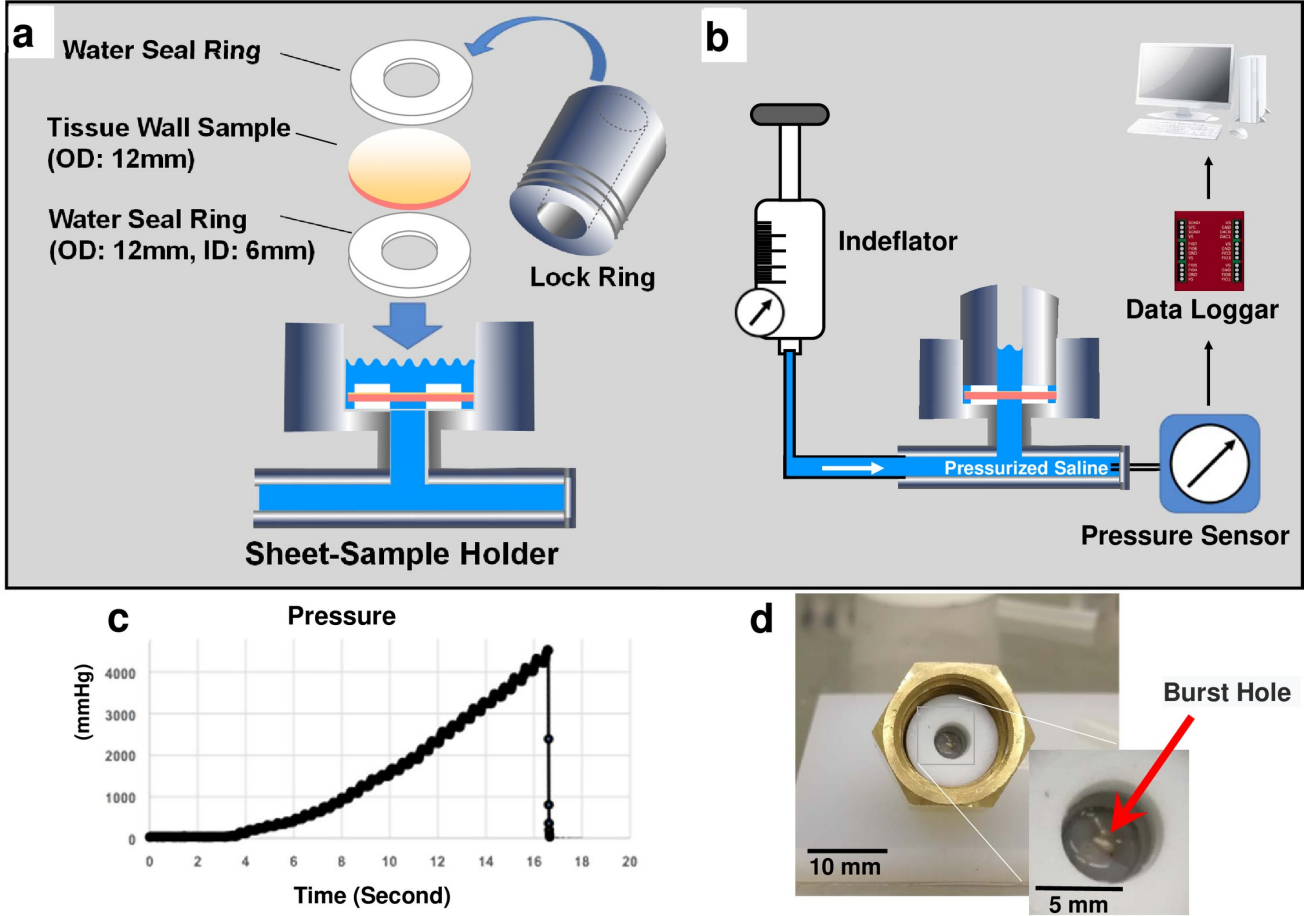
Schematic diagram of a sheet-sample holder (a) and entire circuit appearance (b) for burst pressure measurement. A representative pressure waveform recorded from a ruptured ET sample (c). A representative picture of a ruptured ET sample (d).

Burst pressure tests of the vascular segments were conducted as follows. The segments of tissue tubes were cut open and trimmed to form round membranes with a diameter of 12 mm (n = 18 for each treatment). They were mounted in a sheet-sample folder (Fig 3A) with a hole (6 mm diameter) for pressure loading by saline. A custom pressure measurement circuit (Fig 3B) consisting of a pressurizing inflation device (Indeflator 1000186, Abbott Vascular, CA, USA), a sheet-sample folder, a waterproof digital pressure sensor (PSE560-01, SMC, Tokyo, Japan), and an analog data logger (U3-HV, LabJack Corporation, CO, USA) USB-connected to a computer for the data sampling at a rate of 100 Hz were used. The pressure was increased at a rate of 200−400 mm Hg/sec until failure (Fig 3C). Only the samples which ruptured at a region away from the edge of the folder holes were selected (Fig 3D). ISO7189 instructs that a bursting strength tester with a clamping ring of a diameter such that the area under test is nominally 100 mm^2^ (11.29 mm in diameter). Since we used the sample of 6 mm in diameter, acquired data in our experiment were recalculated to the standard value (0.5316-fold).

Estimated burst pressures were calculated from the UTS values according to the previous study [11] by rearranging the law of Laplace (pressure = 2 [UTS x thickness] / diameter).

### Histological reactions

To evaluate the differences in histological reactions to TEVGs, three groups of chemically treated tissues were re-embedded autologously to the subcutaneous spaces of the same dogs for 4 weeks. Following which, three groups of TEBVs before implantation (n = 2) and after 4-week implantation (n = 2) were histologically evaluated.

The graft specimens were fixed with 4% paraformaldehyde (Wako, Osaka, Japan), embedded in paraffin, sliced into longitudinal sections, and stained with hematoxylin–eosin (HE) and Masson’s trichrome (MT).

### Statistical analyses

The data are expressed as the mean ± standard error (SE). ANOVA and the post-hoc tests (Tukey-Kramer test) were performed using SPSS version 24 (IBM, Chicago, USA). A *p*-value of < 0.05 was considered significant.

## Results

### Macroscopic changes of the tubular tissues before and after chemical modification

**Fig 4:**
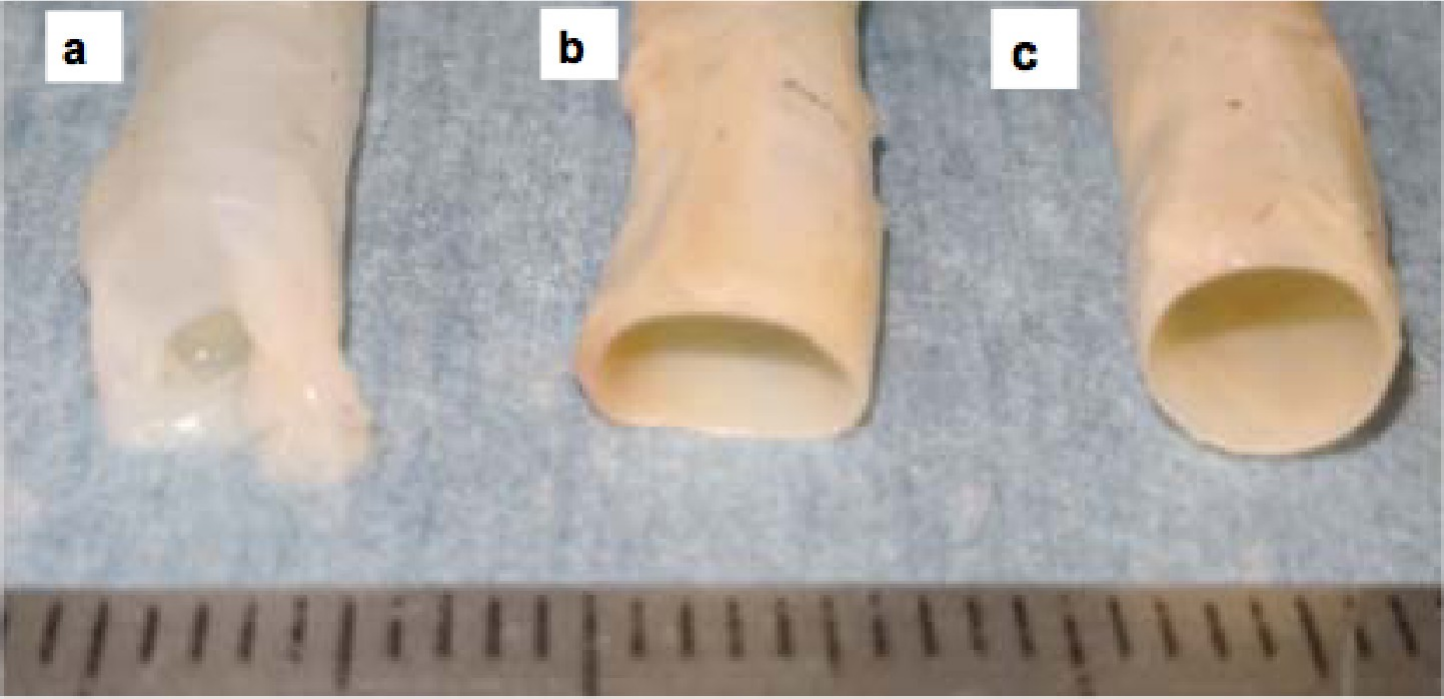
Macroscopic appearance of the grafts before (a), after ET (b), and after GA (c) treatment.

It is very difficult to objectively compare surgical handlings between different types of chemical treatments. However, macroscopically, the RAW group had difficulty in maintaining the cylindrical wall shape. In the air, the tubular tissue collapsed by its own weight, and hence, it was difficult to confirm its lumen during suturing (Fig 4A). In the GA group, however, the cylindrical shape was maintained well due to the impaired flexibility (Fig 4C). In ET group, although a mild distortion of the cylindrical shape was induced by the tissue’s own weight due to its remaining flexibility, it was still easy to confirm its lumen (Fig 4B). The improvements in surgical handling including the suturing test in both ET and GA groups were considered appropriate.

### Vessel ringlet pull test

The UTS was calculated, including the thickness of the specimen acquired from the light microscope measurement (112 ± 21.6 μm).

**Fig 5:**
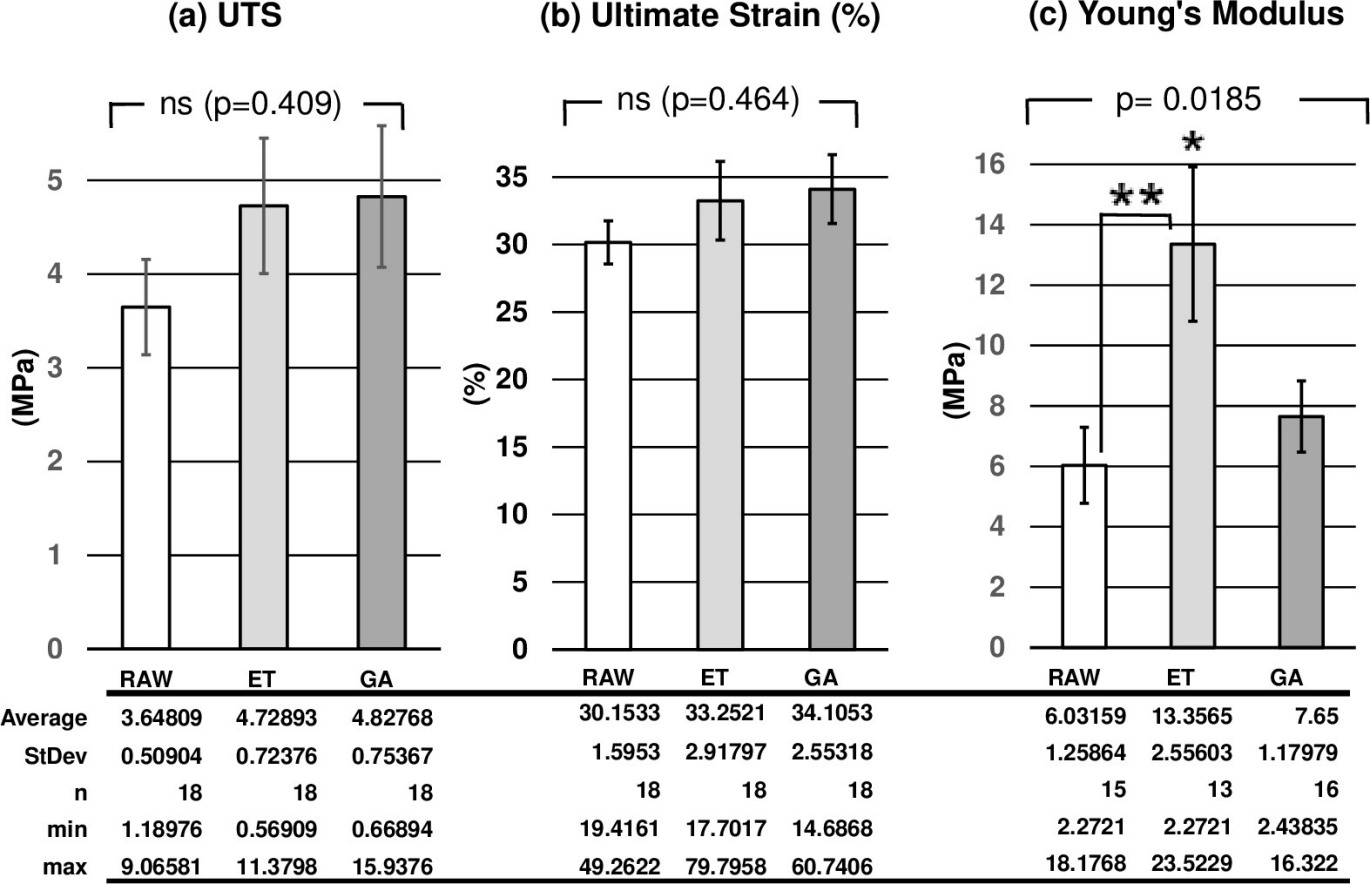
**Results from the vessel ringlet pull test.** Ultimate tensile strength (UTS) (a), ultimate strain (b), and Young’s modulus (c) of the three groups (RAW: RAW group, ET: ET group, GA: GA group, ns: no statistical differences. *Young’s modulus of ET group was significantly higher among the three groups, **Young’s modulus of ET group was significantly higher than that of RAW group.

The UTS of RAW, ET, and GA groups were 3.648 ± 0.509 MPa, 4.729 ± 0.724 MPa, and 4.828 ± 0.754 MPa, respectively. Although the UTS of ET and GA groups tended to be higher, there were no significant differences among the three groups (*p* = 0.409, Fig 5A). The ultimate strain of RAW, ET, and GA groups were 30.15 ± 1.60%, 33.25 ± 2.92%, and 34.11 ± 2.55%, respectively. There were no significant differences among the three groups (*p* = 0.464, Fig 5B). Among the data for calculation of the Young’s modulus, since we could not calculate the value of the slopes from some of the data because of their biphasic waveform. We needed to select 15 data for RAW, 13 for ET and 16 for GA, out of 18 samples. The Young’s modulus of the ET group was significantly higher among the three groups (RAW; 5.41 ± 1.16 MPa, ET; 12.28 ± 2.55 MPa, GA; 7.65 ± 1.18%, *p* = 0.0185). Between the two groups, Young’s modulus of the ET group was significantly higher than that of the RAW group (*p* = 0.0142, Fig 5C).

### Suture retention testing

**Fig 6:**
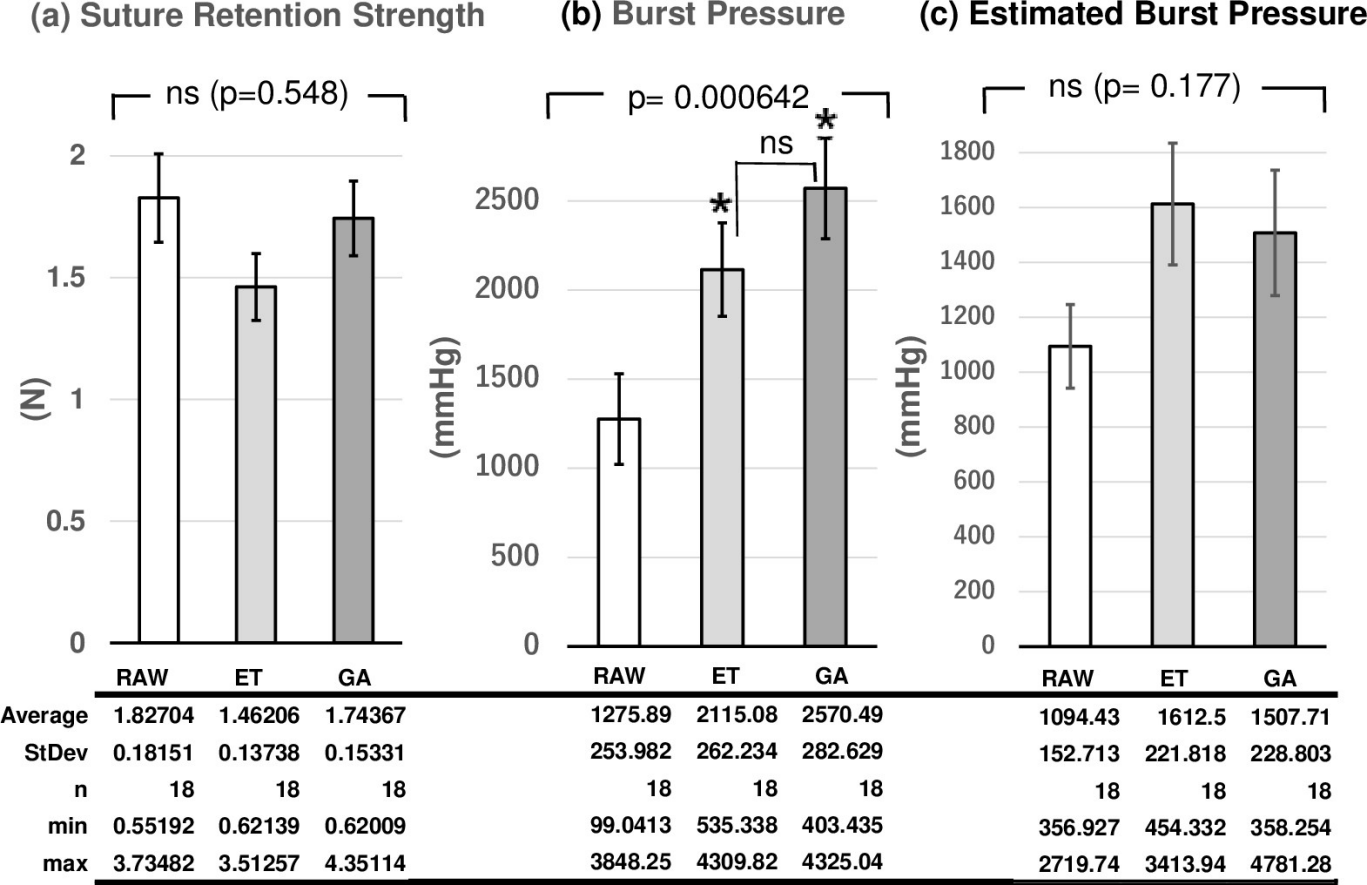
Suture retention strength (a), burst pressure (b) and the estimated burst pressure (c) of the three groups (RAW: group RAW, ET: group ET, GA: group GA, ns: no statistical differences. *Burst pressure of ET and GA groups were significantly higher than that of RAW group).

The suture retention strengths of RAW, ET, and GA groups were 1.696 ± 0.178 N, 1.545 ± 0.168 N, and 1.821 ± 0.186 N, respectively. No significant difference among the three groups was observed (*p* = 0.548, Fig 6A). The minimal suture retention strength of each group was: 0.552 N (RAW), 0.621 N (ET), and 0.620 N (GA).

### Burst pressure measurements

In burst pressure measurements, there were statistical differences among the three groups (*p* = 0.00642, Fig 6B). The burst pressures of ET (2115.1 ± 262.2 mm Hg, *p* = 0.0298) and GA (2570.5 ± 282.6 mm Hg *p* = 0.0017) groups were significantly higher than that of the RAW (1275.9 ± 254.0 mm Hg) group. There was no significant difference in the burst pressure between ET and GA (*p* = 0.247) groups. Minimal burst pressure (RAW; 99.04 mm Hg) was higher in ET (535.3 mm Hg) and GA (403.4 mm Hg) groups. Although estimated burst pressures of ET (1612.5 mm Hg) group and GA (1507.7 mm Hg) group tended to be higher than that of RAW (1094.3 mm Hg) group, the differences were not statistically significant (*p* = 0.1775, Fig 6C).

### Histological reactions

**Fig 7:**
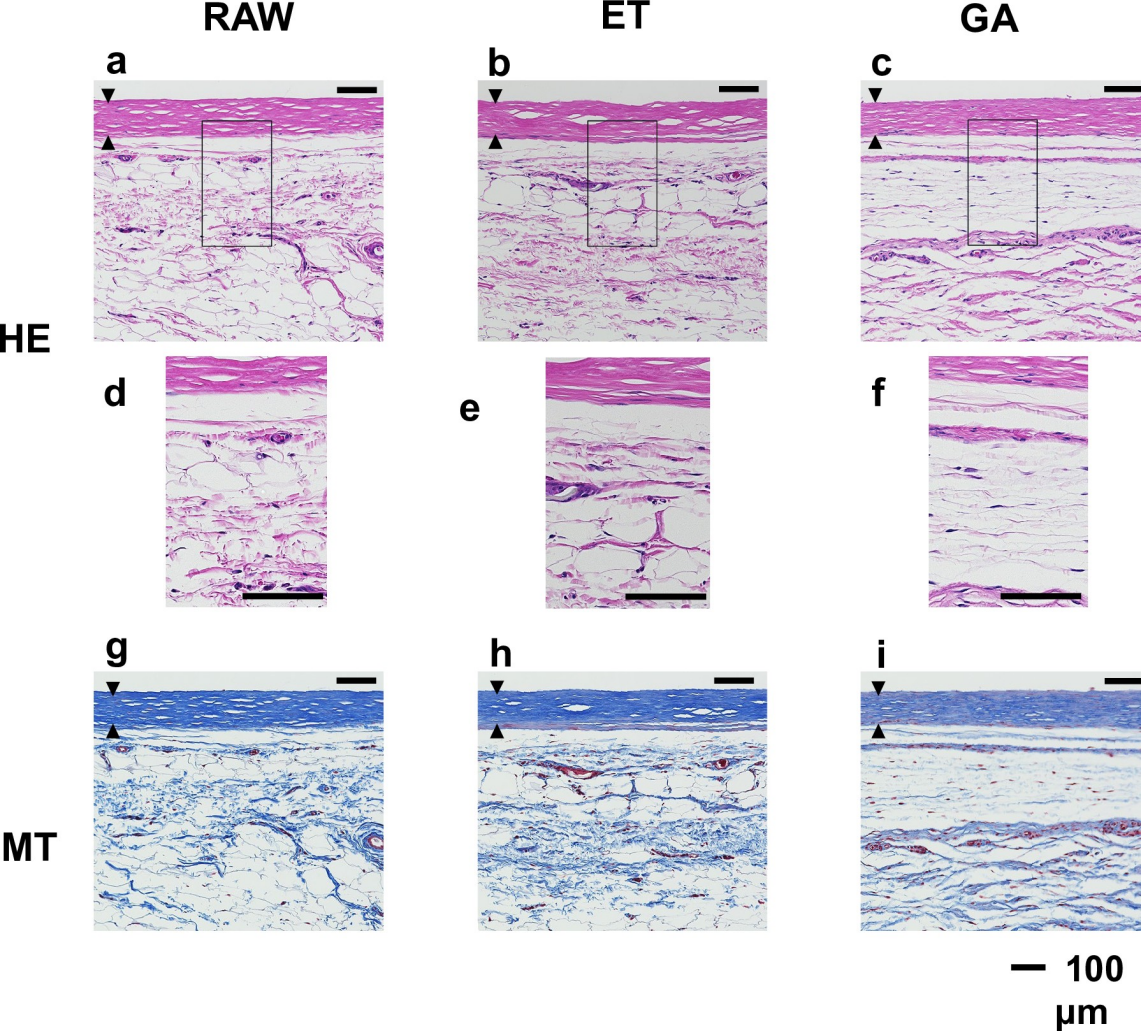
Light microscopy of TEVG after re-implantation to the autologous subcutaneous tissue for 4 weeks (RAW group (a, d, g), ET group (b, e, h) and GA group (c, f, i). HE stain- a, b, c. HE stain (magnified view of the rectangular area in the photo above)- d, e, f. MT stain- g, h, i). TEVGs are indicated between the black triangles. Scale bar, 100 μm. The upper side shows the luminal surface of the TEVG.

The TEVGs were composed mainly of collagen fibers and fibroblasts, with a thickness of 112 ± 21.6 μm. There were no significant differences among the three groups. After re-implantation, no significant inflammatory tissue responses were induced regardless of the 4-week reimplantation in each group (Fig 7). In the higher magnification inserts, no mononuclear cell infiltration was observed at the outer border of TEVGs. There were also no significant differences in the inflammatory responses around the tissues among the groups. Overall, the histological responses in the subcutaneous tissue were inert compared to the tissue infiltration observed when implanted in the vascular systems [6–8]. In addition, the fat tissues around the TEVGs were not damaged which indicated little toxicity of residual chemicals of the grafts.

## Discussion

The Biotube exhibited excellent characteristics as a substitute blood vessel for autologous implantation. However, because formation of tubular tissue strongly depends on the regeneration ability of the host (recipient), the success of Biotube in comorbid patients having extremely poor tissue regeneration activity cannot be guaranteed. In our experience, the skin of the premature infant is extremely thin and their subcutaneous space for the mold impregnation was narrow and limited. Further wounds in high-risk patients, including those with diabetes, are typically slow to heal. Thus, tissue formation will likely be retarded in such patients. Therefore, improvement in mechanical durability of grafts is important in order to avoid mechanical problems after implantation, such as rupture or pseudoaneurysm formation. To this end, several kinds of approaches to form stronger tissue grafts have been developed. One possible option is the development of the mold design to form the more robust tissues, such as mold surface modification by etching [12] or by using a two-layered cage mold [13]. Chemical pretreatments, such as dehydration by ET or crosslinking by GA are the other techniques attempted to improve the mechanical properties. To our knowledge, no studies that quantitatively describe the differences in mechanical properties of the Biotubes after different chemical treatments have been reported; therefore, we performed several kinds of mechanical tests in this study. Macroscopic evaluation and actual surgical handling tests, including suturing procedures, showed that both ET and GA treatments improved surgical handling, despite the difficulty of an objective evaluation. Accordingly, ET dehydration was selected for the clinical application to improve surgical handling [9].

In this experiment, we evaluated the advantages of chemical treatments pertaining to both subjective surgical handling and objective mechanical properties. The mean burst pressures of RAW, ET, and GA groups (ID: 5 mm) were 1275.9, 2115.1 and 2570.5 mmHg, respectively. The burst pressures of the native arteries were reported in previous studies [14, 15]. Among them, the gold standard is that of human saphenous veins (SV), the burst pressure of which was about 1680 mmHg [14]. Burst pressure of another native vessel, such as an internal mammary artery (IMA), was 3196 mmHg [15]. The burst pressure of other TEVGs constructed from human cells, which had already undergone clinical applications were reported to be 3490 mmHg (ID: 4.8 mm) [15], 2594 mmHg (ID: 4.6 mm) [14], and 3337 mmHg (ID: 6 mm) [16]. Each TEVG in these studies was developed to achieve the burst pressure equivalent to that of the human SV. The average burst pressure in this study was significantly increased in the ET and GA groups and exceeded that of the human SV while that of RAW group was lower. Moreover, because the values fluctuated widely, minimal burst pressure of the RAW group was not considered high enough for the use in arterial pressure systems. For example, the minimum burst pressure in randomly selected areas of the RAW group was only 99.04 mmHg, which was below the normal systolic arterial pressure, whereas the same in both ET (535.3 mmHg) and GA (403.4 mmHg) groups were high enough. These results indicate that higher initial mechanical durability for pressure loading before implantation was achieved by ET and GA treatments.

Unlike burst pressure measurement, there was no significant difference among the three treatment groups in the suture-retention strength test (RAW; 1.696, ET; 1.545, GA; 1.821 N). Suture retention strength of the human native peripheral vessel with similar diameters were also reported in the previous study (SV; 196 gf = 1.92 N, IMA;138 gf = 1.35 N) [15]. In the other clinically applied TEVGs, results were reported as 152 gf = 1.49 N [14] and 178 gf = 1.75 N [16]. Both of these TEVGs were aimed to achieve the equivalent or more strength of the native vessels shown above. Our grafts were also as strong as native vessels. The minimal values of our study in RAW, ET, and GA groups were 0.552 N, 0.621 N, and 0.620 N, respectively, all of which exceeded the lot release criteria of Konig et al. [15], who established it as 50 GMF (= 0.490 N) for tissue-engineered vascular grafts to be used in clinical trials. Therefore, our *in vivo* tissue-engineered vascular grafts satisfied the safe condition regardless of the chemical treatment.

We conducted a tensile stress test via a vessel ringlet pull test because it is widely used for evaluating many other kinds of tissue-engineered vascular grafts and is also described in ISO 7198 [10]. The results obtained by this method were comparable to those reported by other research groups [11, 12, 15]. The results of UTS were also converted to the theoretical burst pressure values using Laplace’s relationship [11]. UTS acquired from this study was converted to the estimate burst pressure (RAW: 1094.4 mmHg, ET: 1612.6 mmHg, GA: 1507.7 mmHg; Fig 6C). The estimated burst pressure of the clinically applied TEVG shown above [15] was 4734 mmHg whose actual pressurized burst pressure was 3196 mmHg. In the other TEVG estimated burst pressure was reported to be 3947 mmHg (ID: 4.2 mm) [12] and 2127 mmHg (ID: 5.5 mm) [11], which were comparable to that of our TEVG (ID: 5 mm). In this study, there were no significant differences in estimated burst pressure among the different treatments because UTS was not altered by chemical treatment. Although the precise reason of the significant increase of the value from only the direct burst pressure measurement by the two treatments is unclear, we hypothesize that it is because of the extremely sensitive nature of the direct burst pressure test. UTS from vessel ringlet pull test reflected the mean value of the entire wall strength of the 5 mm-long tubular tissue. Further, the direct burst pressure testing in this study indicated the minimal wall strength of the entire circular area with a diameter of 6 mm. The minimal estimated burst pressure of the RAW group (356.9 mmHg) was much higher than that of the pressurized burst pressure (99.04 mmHg), which might make us miss the weak TEVG during mechanical testing. Therefore, direct burst pressure measurement might be more practical to assess the mechanical reliability than the theoretical burst pressure calculated from the UTS value. Recently, indirect burst pressure and direct burst pressure of synthetic material tubes, native vessels, and TEVGs were compared [17]. They noted that indirect burst pressure obtained from UTS was well correlated to the direct burst pressure in the synthetic materials. However, because biological materials, such as native vessels and regenerated TEVG, were not homogeneous like artificial materials, correlation between the direct burst pressure and the theoretical burst pressure was not sufficient for the assessment of clinical applications. In our direct burst pressure measurement, we used the small seat materials (diameter; 6 mm) incised from the tubular grafts different from the standard method using the whole length of tubular grafts because we often could use very limited test samples in the clinical conditions, especially when they were constructed in the small children.

From five kinds of mechanical property tests, the only concern regarding the chemical treatment was the increase in Young’s modulus by ET treatment signifying the reduction in tissue compliance. The higher initial compliance mismatch of the ET-treated tissues as compared to that of the raw tissues might cause anastomotic problems, such as stenosis due to excessive intimal hyperplasia at the anastomosis site [18, 19]. Young’s modulus of the native vascular tissues varies widely. For example, those of human umbilical artery, vein and aorta were 0.4 MPa, 1.8 MPa, and 3 MPa [20], respectively. Those of porcine aorta, vena cava, and carotid artery were 1.9, 6.9 and 1.5 MPa, respectively [21]. However, the values of Young’s modulus in our ET-treated vascular tissues (13.35 MPa) were much lower than those of the native pericardia (20–50 MPa, [22, 23]), which are widely used for reconstruction of the cardiovascular tissues [24]. In fact, our ET-treated *in vivo* tissue-engineered vascular tissue implanted to the pulmonary artery of a 9-month old child was not found to induce any stenotic changes [9]. Specifically, no inferiority of ET or GA treatment was detected in any of the five kinds of mechanical properties tests in this study. Accordingly, we could aggressively adopt chemical treatments from both the perspectives of improved macroscopic surgical handling and burst pressure.

In the morphological evaluations, contrary to the macroscopic changes (Fig 4A–4C), there were no significant differences in the light microscopic level among the three groups. We evaluated the histological responses to the chemically treated TEVGs, which were autologously implanted to the subcutaneous tissues of the same beagle dogs, and showed that short periods of chemical treatment using ET and GA did not appear to interfere with the histological responses due to excessive inflammatory or toxic activities (Fig 7). From the viewpoint of toxicity, GA has high sustainability and is difficult to rinse thoroughly as compared to ET [25]. In addition, clinical reports have highlighted that GA toxicity lasts for a longer period [26, 27]. Anastomotic problems due to tissue destruction by the toxicity of bovine serum albumin and glutaraldehyde glue was exhibited [26]. Furthermore, GA-treated tissue grafts induce calcification reaction after transplantation [27]. Therefore, in the present clinical study, we selected ET for standard pretreatment of the vascular grafts in terms of surgical handling, strength, and reduced toxicity.

## Conclusion

There were some limitations to this study. First, we evaluated only one representative clinically used treatment condition for ET [28] and GA [29], and the effects of other conditions of treatment with ET and GA, such as different concentrations, temperatures, and durations, should be evaluated to determine the ideal condition. Chemical modification is just one of the possible options to improve the mechanical properties of the grafts. To conclusively overcome the issue of the individual variety of the tissue regeneration *in vivo*, we need to investigate additional options. We also began another approach using more reliable tissues derived from healthy individuals via allogeneic or xenogeneic transplantation [30]. Second, further investigation of their long-term biological performance is very important. We will continue to follow the histological changes of our clinically implanted TEVGs to evaluate their biocompatibility.

Chemical treatment by ET and GA on *in vivo* tissue-engineered vascular grafts could improve surgical handling and burst pressure tolerance and successfully modify their mechanical properties without interfering with tissue regeneration.
